# Ostracism, Psychological Capital, Perceived Social Support and Depression among Economically Disadvantaged Youths: A Moderated Mediation Model

**DOI:** 10.3390/ijerph182111282

**Published:** 2021-10-27

**Authors:** Xianglian Yu, Lin Zhang, Zihong Lin, Zongkui Zhou, Dilana Hazer-Rau, Pinlin Li, Wenlong Ji, Hanbing Zhang, Tong Wu

**Affiliations:** 1 School of Psychology, Central China Normal University, Wuhan 430056, China; psyyu@jhun.edu.cn (X.Y.); zhouzk@ccnu.edu.cn (Z.Z.); wutong2019@mails.ccnu.edu.cn (T.W.); 2 Department of Education, Jianghan University, Wuhan 430056, China; lipinlinnew@outlook.com (P.L.); 182203301212@stu.jhun.edu.cn (W.J.); ZhangHanbing@stu.jhun.edu.cn (H.Z.); 3 Key Laboratory of Adolescent Cyberpsychology and Behavior, Ministry of Education, Wuhan 430056, China; 4 Key Laboratory of Human Development and Mental Health of Hubei Province, Wuhan 430056, China; 5 Student Affairs Department, Fujian Normal University, Fuzhou 350007, China; zihonglin@fjnu.edu.cn; 6 Section of Medical Psychology, Department of Psychosomatic and Psychotherapy, University of Ulm, 89075 Ulm, Germany; dilana.hazer@uni-ulm.de

**Keywords:** ostracism, depression, psychological capital, perceived social support, economically disadvantaged youth

## Abstract

Mental health promotion of economically disadvantaged youths is a popular issue in current China. Economically disadvantaged youths are at greater risk of depression. Ostracism may be an important predictor of depression for them. However, no consensus has been reached on the underlying mechanism between ostracism and depression. A total of 1207 economically disadvantaged youths were recruited from six universities in China. These youths were asked to complete questionnaires measuring depression, ostracism, psychological capital, and perceived social support. A moderated mediation model was examined by using IBM *SPSS* STATISTICS 27macro program PROCESS version 3.5, in which psychological capital was a mediating variable, and perceived social support was a moderating variable. Lack of causal inferences and self-report bias due to the cross-sectional and self-report survey need to be considered when interpreting results. The results revealed that ostracism was positively associated with depression among economically disadvantaged youths. Psychological capital partially mediated the association. Perceived social support moderated the indirect association between ostracism and depression via psychological capital among economically disadvantaged females. Training and intentional practice of psychological capital could be the core to develop the depression interventions targeting economically disadvantaged youths with experience of ostracism. Gender and perceived social support need to be considered in developing the interventions.

## 1. Introduction

Depression is a significant public health issue, with a prevalence of 3.6% among youths in China [1]. Youths from economically disadvantaged backgrounds are at greater risk of depression [2,3]. For society, depression is not only a leading cause of disability but also a major contributor to the global burden of disease. For individuals, depression leads to a decline in social functioning, sleep and appetite disturbances, and suicide attempts, etc. [4]. Therefore, identifying variables that may cause depression or contribute to a reduction in economic inequalities in depression is important to solve the problem of mental poverty and consolidate the success of poverty eradication in China [5].

Ostracism, as one of the predictors of depression, has become of great concern in the field of research on economically disadvantaged youths. First, the negative effects of ostracism are especially profound for youths [6]. They are sensitive to social stimuli and the negative effects of social exclusion at this age [7]. Second, economically disadvantaged individuals may be more prone to perceive inequalities and be ostracized [8,9]. However, some researchers proposed that prior experiences of adversity during adolescents may strengthen resistance to later stress, which is beneficial to individuals’ survival and psychological regulation [10]. In order to consolidate the success of economic poverty eradication in China, the Chinese public may pay more attention to mental poverty and relevant social problems [11,12]. Hence, investigating the effect of ostracism on depression and the underlying mechanism is of significance.

Therefore, in the present study, we investigated the association between ostracism and depression among economically disadvantaged individuals, as well as examined a moderated mediation model of this association. Specifically, we first investigated the mediating role of psychological capital (PsyCap) in the link between ostracism and depression. Furthermore, we examined the moderating role of perceived social support in the mediation model.

### 1.1. Ostracism and Depression

Ostracism is defined as ignoring or excluding individuals by other individuals or groups [13]. Both theoretical and empirical studies support the potential effect of ostracism on depression among economically disadvantaged individuals. Specifically, according to social causation theory, Patel and Kleinman [14] believed that ostracism or social exclusion rather than income level was a major reason for depression among economically disadvantaged individuals. Moreover, Allen and Badcock [15] proposed a social risk theory, which suggested that ostracized individuals perceived that they were the burden of others and valueless to others. For these ostracized individuals, depression is a functional illness that keeps them from engaging in social interactions.

Empirical studies also revealed the association between ostracism and depression. Many previous cross-sectional investigations among Chinese youths provided strong evidence for this relationship [16,17]. Besides, some previous longitudinal studies also suggested that ostracism could predict depression symptoms among youths, although these studies did not control for other contextual factors [18,19]. Moreover, a large number of experimental studies revealed that ostracism induced negative emotional responses among youths across experimental paradigms [20]. However, on the contrary, some researchers believed that ostracism would elicit emotional reactions but not cause distress nor depression [21]. The determinant for depression was rejection sensitivity or how individuals remembered the experience of ostracism [22,23].

### 1.2. Psychological Capital as a Mediator

Psychological capital (PsyCap), characterized by self-efficacy, optimism, hope, and resilience [24,25], is not only a positive psychological state that develops throughout the process of growth and development [25], but also a psychological resource that helps individuals handle the environment and themselves [26].

No consensus has been reached on the role of PsyCap in the association between ostracism and negative consequence. Several researchers considered PsyCap to be a moderator between workplace ostracism and negative emotion or behaviors [27,28]. However, as a state-like variable, a majority of researchers believed that ostracism during childhood would have great significance for PsyCap. PsyCap falls in between transient states and “hard-wired” traits, which suggests that it can be changed by long-standing environmental factors and improved by training and intentional practice [24]. A previous study found that rejection as one of the early maladaptive schemas could be negatively related to an individual’s PsyCap [29]. According to the temporal framework, ostracism elicits reflexive painful responses immediately, and then threatens psychological needs and induces negative emotions, and then induces cognitive appraisals. That is, efficacy and existence needs, as the second phase in the response to ostracism, can be threatened by ostracism [30]. Experimental studies also found that the ostracism group had a lower level of resilience or self-efficacy compared with the social inclusion group [31,32]. Because self-efficacy, optimism, hope, and resilience are components of PsyCap, it is reasonable to assume that ostracism may decrease the level of PsyCap.

Moreover, PsyCap can predict depression. According to the broaden-and-built theory, psychological resources (e.g., PsyCap) can help individuals form positive emotion regulation strategies against negative emotions [33]. Hobfoll [26] proposed the conservation of resources theory, which suggested that psychological resources could promote youths to pursue well-being, even when these youths were under the pressure of life. Previous cross-sectional investigations can also provide evidence for the association. For example, a negative association between PsyCap and depression was found in an investigation among depressed adolescents in China [34]. Besides, previous clinical studies found that psychological capital intervention (PCI) was effective to increase PsyCap and then decrease depressive symptoms [35]. These theoretical bases and empirical evidence revealed that PsyCap could be a predictor of depression. Taken together, we can assume that PsyCap is a mediating variable in the association between ostracism and depression.

### 1.3. Perceived Social Support as a Moderator

As mentioned above, ostracism may decrease the level of PsyCap. However, not all economically disadvantaged youths who have an experience of ostracism decrease equally in their levels of PsyCap, in which perceived social support might play an important role [36]. Perceived social support is conceptualized as individuals’ perception of supportive events, which can be considered as one of the socialization resources. Perceived social support is different from received social support because the latter is a reflection of actual support provided by the environment but the former is more concerned with the perception [37]. Empirical studies found socialization resources had a positive relationship with PsyCap [38]. Moreover, previous studies also found that both perceived social support and organizational climate could affect the PsyCap [39]. One possible reason is that the social support perceived by a person exposed to adversities is crucial for sustaining resilience. As a socialization resource, perceived social support may enhance the components of PsyCap [40].

Regarding how perceived social support moderates the association between ostracism and PsyCap, there has been disagreement between researchers thus far. Some researchers believed that adolescents probably developed an ability to deal with ostracism if they were in a situation where they perceived little social supports for a long time because ostracism might be a piece of their life that lacks support. That is, the association between ostracism and PsyCap may be weak in this situation. When adolescents perceived strong social support, ostracism would become a major and memorable event, which would be negatively associated with PsyCap and further positively associated with depression [23]. On the contrary, other researchers considered that perceived social support would be a protective variable, which indicated that the association between ostracism and PsyCap would be weaker if individuals perceived higher social support than lower social support [38]. Therefore, it is necessary to examine whether perceived social support would moderate the indirect association between ostracism and depression via PsyCap among economically disadvantaged youths. If yes, how does the perceived social support moderate the association?

### 1.4. Present Study

**Figure 1:**
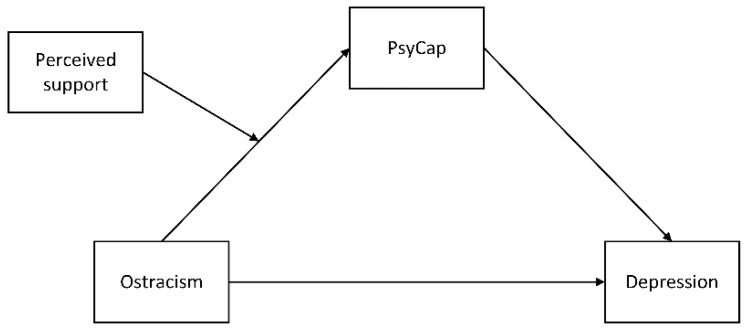
The proposed moderated mediation conceptual model.

Although previous studies have proven the positive relationship between ostracism and depression, few studies examined this relationship among economically disadvantaged youths. Besides, the underlying mechanism in the link between ostracism and depression has not been fully investigated. On the basis of theoretical and empirical studies mentioned above, the present study aimed to (1) examine the association between ostracism and depression among economically disadvantaged youths, (2) investigate whether PsyCap could mediate the association between ostracism and depression, (3) examine whether perceived social support would moderate the mediating effect of PsyCap in the relationship between ostracism and depression. Therefore, we proposed the following hypotheses (the proposed moderated mediation conceptual model is shown in Figure 1):

**Hypothesis** **1.** *Ostracism would be positively associated with depression among economically disadvantaged youths.*

**Hypothesis** **2.** *PsyCap would mediate the association between ostracism and depression, that is, ostracism would be negatively associated with PsyCap, and PsyCap would be negatively associated with depression.*

**Hypothesis** **3.** *Perceived social support would moderate the indirect effect of ostracism on depression via PsyCap. The negative association between ostracism and PsyCap is moderated by perceived social support, such that it is stronger for higher than for lower levels of perceived social support.*

## 2. Methods

### 2.1. Participants and Procedure

A total of 1207 college students from economically disadvantaged families were recruited at two universities in Central China and four universities in Southeast China. The identification of students from economically disadvantaged families was based on the criteria set by each province. Specifically, they had to meet at least one of the following criteria: (1) students from economically disadvantaged families confirmed by the poverty alleviation department, (2) students from economically disadvantaged families confirmed by the civil affairs department, (3) orphans, de facto unsupported children confirmed by the civil affairs department, (4) children of martyrs with financial difficulties confirmed by the Veterans Affairs Department, and (5) students with disabilities and financial difficulties confirmed by the department of the Federation of Disabled Persons. A total of 1207 questionnaires were collected through an online platform. The participants included 907 females (75.1%) and 300 males (24.9%) and consisted of 221 freshman (18.3%), 189 sophomores (15.7%), 512 juniors (42.4%) and 285 senior students (23.6%). The average age of the sample was 17.21, with a standard deviation of 2.62.

All participants gave their consent to participate in the study and allowed their data to be used for the research. Data collection was anonymous and voluntary. The questionnaire information was kept confidential. The study was approved by the Life Science Ethics Committee of Central China Normal University.

### 2.2. Measures

#### 2.2.1. Outcomes

The depression severity was measured using the Chinese version of the Zung Self-Rating Depression Scale (SDS) [41]. The SDS is a 20-item, self-report, and 4-point Likert scale (1 = a little of the time to 4 = most of the time), including affective, psychological, and somatic symptoms associated with depression [42]. A high total score indicates severe depressive symptoms. In the current study, the internal consistency was good (Cronbach’s α = 0.814).

#### 2.2.2. Predictor Variable

Ostracism was measured using the Chinese version of the Ostracism Experience Scale for Adolescents (OES-A) [17,43]. The OES-A is an 11-item, self-report, and 5-point Likert scale (1 = never to 5 = always), including Excluded and Ignored subscales [44]. The OES-A Excluded subscale measures an adolescent’ perceptions of being socially rejected by peers (e.g., “*In general, others include me in their plans for the holidays*”), while the OES-A Ignored subscale measures an adolescent’ perceptions of being socially neglected by peers (e.g., “*In general, others ignore me during conversation.*”). Higher total scores of (sub)scales reflected higher levels of perceived ostracism, excluded or ignored. In the present study, the internal consistency for the OES-A was good (Cronbach’s α = 0.815).

PsyCap was measured using the Positive Psycap Questionnaire (PPQ) (Zhang et al., 2010). The PPQ is a 26-item, self-report, and 7-point Likert scale (1 = strongly disagree to 7 = strongly agree), including four subscales, self-efficacy (7 items), optimism (6 items), hope (6 items), and resilience (7 items). The self-efficacy subscale measures an individual’s belief in his or her capacity to shoulder and put in the necessary effort to conquer challenging tasks (e.g., “*I am willing to take on difficult and challenging work*”). The optimism subscale measures an individual’s positive attribution of success now and in the future (e.g., “*I pursue my goals with confidence*”). The hope subscale measures a state of persevering or redirecting paths to goals in order to succeed (e.g., “*I always look on the bright side of things*”). The resilience subscale measures an individual’s ability to sustain adversity and bounce back from difficult life events (e.g., “*I can bounce back quickly when I encounter setbacks*”). The scale has been widely used to measure the psychological capital in China and demonstrated good internal consistency and validity [34,45]. In the present study, the internal consistency for PPQ was good (Cronbach’s α = 0.931).

Perceived social support was measured using a Chinese version of the Multidimensional Scale of Perceived Social Support (MSPSS) [43,46]. MSPSS is a 12-item, self-report, 7-point Likert scale (1 = strongly disagree to 7 = strongly agree). It consists of three subscales, namely, social support from family (e.g., “*My family really tries to help me*”), friends (e.g., “*My friends really try to help me*”), and significant other (e.g., “*There is a special person who is around when I am in need*”). Higher total scores indicate stronger social support. In the present study, the internal consistency of the MSPSS was good (Cronbach’s α = 0.957).

### 2.3. Statistical Analyses

Data analyses were carried out using SPSS version 27.0 software and the SPSS macro program PROCESS version 3.5 (Andrew F. Hayes, Calgary, Canada) [47]. First, we conducted descriptive analyses and correlation analyses. Then, all continuous variables were mean-centered. Next, we selected Model 4 to assess the mediating effect of PsyCap between ostracism and depression and selected Model 7 to assess the effect of moderated mediation, with gender as covariates. Bias-corrected percentile bootstrap method was used and 95% confidence intervals (CI) with 5000 resampled samples were calculated for testing of theoretical hypothesis. If the 95% CI did not include 0, it meant that the statistics were significant. Notably, the cross-sectional and self-report survey used in the present study would bring limitations such as lack of causal inferences, recall bias, and social desirability bias.

## 3. Results

### 3.1. Descriptive Analyses and Correlation Analyses

Table 1 shows the descriptions and correlations of all variables in the study. As we expected, ostracism was positively correlated with depression (r = 0.623, *p* < 0.001). PsyCap (r = −0.581, *p* < 0.001) and perceived social support (r = −0.383, *p* < 0.001) were negatively correlated with depression. In addition, ostracism was negatively correlated with PsyCap (r = −0.526, *p* < 0.001) and perceived social support (r = −0.436, *p* < 0.001). PsyCap was positively correlated with perceived social support (r = 0.584, *p* < 0.001).

### 3.2. Test of Mediation

Model 4 of the SPSS macro PROCESS was utilized to test the mediating effect of PsyCap on the relationship between ostracism and depression while controlling for gender. Table 2 shows the result of the mediation analysis. Specifically, the positive predictive effect of ostracism on depression was significant (B = 0.74, t = 27.51, *p* < 0.001). The negative predictive effect of ostracism on PsyCap was significant (B = −1.53, t = −21.85, *p* < 0.001), while PsyCap had a significant negative predictive effect on depression (B = −0.15, t = −14.50, *p* < 0.001). Furthermore, the direct predictive effect of ostracism on depression was significant when the mediating variable was added (B = 0.51, t = 17.49, *p* < 0.001). The 95% CI of bias-corrected percentile bootstrap for the direct effect of ostracism on depression and the mediating effect of PsyCap did not include 0, which means the mediating effect was significant (indirect effect = 0.23, SE = 0.003, 95% CI = (0.19, 0.27)). The mediating effect accounted for 30.6% of the total effect, indicating that the PsyCap played a partial mediating role in the ostracism–depression link.

### 3.3. Test of Moderated Mediation

**Figure 2:**
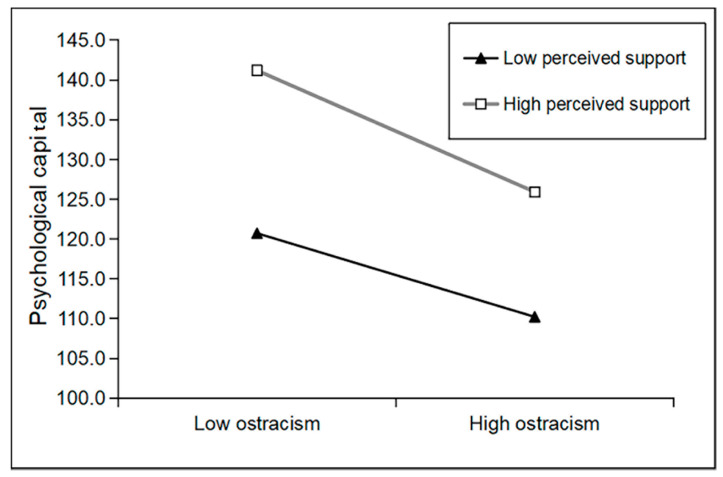
Perceived social support moderates the effect of ostracism on psychological capital.

Table 3 shows the results of the moderated mediation analysis. The interaction term between ostracism and perceived social support was negatively related to psychological capital (B = −0.014, t = −2.949, *p* = 0.003). In addition, simple slope analysis showed that for youths with stronger social support, higher ostracism was negatively associated with psychological capital (B = −1.111, t = −13.587, *p* < 0.001). However, for youths with weaker social support, the relationship between ostracism and psychological capital was not significant (B = −0.361, t = −1.643, *p* = 0.101) (See Figure 2).

Moreover, we also investigated the gender effects of the hypothesized model. The results can be seen in the Supplementary Material File S1. The only different pattern was the moderated mediation effect among male participants. The effect of the interaction term (ostracism × perceived social support) on PsyCap was not significant (B = −0.01, t = −0.51, *p* = 0.61) in the male participants. Except for the above effect, male, female and the whole sample shared the same pattern of results.

## 4. Discussion

The present study examined the relationship between ostracism and depression among economically disadvantaged youths and investigated the possible mediating role of PsyCap and moderating role of perceived social support underlying the relationship. Our results show that ostracism was positively associated with depression, PsyCap partially mediated the link of ostracism–depression, and perceived social support moderated the indirect path between ostracism and depression. These findings may help researchers and practitioners to better understand how ostracism affects depression among economically disadvantaged youths and further develop intervention programs for depression specific to these youths.

### 4.1. The Mediating Role of Psychological Capital

Our results indicate that a high level of ostracism was associated with a high level of depression, which is not only consistent with previous studies [15], but also can be supported by the social risk theory [20,48]. Specifically, a large number of cross-sectional and longitudinal studies have found that ostracism predicts depression among adolescents [20,49]. Experimental studies have also found a reciprocal relationship between experimental ostracism and depression-like symptoms [15]. This finding can be explained by the fact that long-lasting excluded persons are not willing to take the risk to engage in social interaction, and depression is a functional response that allows them to avoid taking this risk [48,50]. One of the contributions of the present study is to verify this association among economically disadvantaged youths, which extends the generalizability of the association to economically disadvantaged youths.

Moreover, our results showed ostracism not only directly predicted depression but also indirectly affected depression through the mediating variable of PsyCap among economically disadvantaged youths. PsyCap has not been examined as a mediating variable in the link of ostracism–depression, although previous studies have investigated some mediating variables between ostracism and depression [13]. No direct evidence for the mediating role has been found; however, previous studies have found that ostracism threatens basic psychological needs, such as self-esteem, self-efficacy, control, and meaningful existence [34], which may impair the state-like PsyCap further. Furthermore, empirical studies have reported that PsyCap reduces the risk of depression [34]. Taken together, there is strong evidence to support our results that ostracism predicts depression through PsyCap.

We also found that the effect size of ostracism on depression among economically disadvantaged youths in China was moderate to large, which was larger than that among adolescents in the general population, which indicates that the negative consequence of ostracism may be larger for economically disadvantaged youths [17]. Fortunately, we found that the effect size of PsyCap on depression among economically disadvantaged youths was moderate, which was also larger than that among adolescents in the general population, which implies that economically disadvantaged youths can develop effective strategies to deal with their disadvantaged living situations [34].

### 4.2. The Moderating Role of Perceived Social Support

The present study also examined the moderating role of perceived social support in the mediation model. To the best of our knowledge, this is the first study to examine the moderating role of the perceived social support in the indirect association between ostracism and depression via PsyCap. Our results showed that perceived social support moderated the association between ostracism and PsyCap. For economically disadvantaged youths with a higher level of perceived social support, the influence of ostracism on PsyCap was stronger than that for youths with a lower level of perceived social support. That is, economically disadvantaged youths who reported a relatively low level of ostracism and perceived a high level of support had the highest level of PsyCap. Similarly, economically disadvantaged youths reported a relatively high level of ostracism but perceived a high level of support had a higher level of PsyCap. However, individuals who perceived a low level of support reported lower PsyCap, whether they experienced ostracism or not.

Consistent with previous studies [36,51], our results indicated that the adverse effects of low perceived social support were significant, regardless of whether economically disadvantaged youths were ostracized. A possible explanation is that individuals who lack the ability of processing pleasure and social acceptance are more difficult to develop psychological resources and more vulnerable to mental illness. Specifically, a previous study found that depressive individuals not only showed lower scores on positive affect but also decreased P3 amplitudes in response to social acceptance compared to healthy control individuals, which indicated that the reason for depression may be the dull to social acceptance/support and the lack of psychological resources motivated by socialization resources [51]. Moreover, our results also showed that a higher level of ostracism predicted a lower level of PsyCap if economically disadvantaged youths perceived a high level of social support, which can be explained by that for individuals who are sensitive to both positive and negative cues, PsyCap is impacted by the interaction of them [38].

Remarkably, our results showed that the perceived social support moderated the association between ostracism and PsyCap among economically disadvantaged female youths only. Among economically disadvantaged male youths, the moderating effect was not significant. That is, our model is a conditional moderated mediation model. These results are consistent with previous studies in which female youths value social support more and are more sensitive to the lack of social support compared with male participants due to differences in evolutionary outcomes, socialization, and motivations across sexes [52,53].

### 4.3. Implications

The present study has at least three theoretical contributions and practical implications. First, a theoretical implication of the study is that the moderated mediation model complements the social causation theory and the social selection theory. Specifically, both theories emphasize that ostracism leads to an increased risk of depression among economically disadvantaged individuals [12]. However, these theories did not reveal the underlying mechanism of the association between ostracism and depression, that is they did not investigate the potential mediating or moderating variables in the link of ostracism and depression. As a useful addition to these theories, the present study investigated the mediating role of PsyCap and the moderating role of perceived social support.

Second, previous studies have focused on the maladaptive behavior-related variables (e.g., self-control) in the ostracism–depression link [50], but have tended to overlook the psychological resource-related variables from the perspective of positive psychology. As a state-like variable, PsyCap is positive-oriented and can be developed through practice and training This study provides an insightful perspective to reach a better understanding of how human strengths lessen the adverse consequence of ostracism and reduce the risk of depression.

Last but not least, the findings of the present study may provide the following practical implication. As PsyCap is an important mediating variable in the link between ostracism and depression, researchers and practitioners should consider methods and strategies for improving PsyCap and incorporate them into the intervention on depressive symptoms targeting economically disadvantaged youths with an experience of ostracism. Besides, it is necessary to add elements for promoting the sensitivity to social support in the design of depression intervention programs. Furthermore, careful consideration should always be given with regard to gender differences in the intervention programs.

### 4.4. Limitations

Notwithstanding the above contributions and implications, several limitations have to be admitted regarding the present study. First, the causal association between ostracism, PsyCap, and depression cannot be determined by the present cross-sectional design. Further longitudinal studies need to be undertaken to validate the moderated mediation model. Moreover, further experimental studies should focus on determining the causality between ostracism and PsyCap, because some experimental studies have found that the experience of ostracism precedes negative affect, and the change in PsyCap precedes changes in depressive symptoms.

The second limitation of the present study is the potential issue of the representativeness of the sample, as females are more than males in our sample. Although some researchers have reported that more economically disadvantaged females are more than males in their studies [54,55], we cannot say economically disadvantaged females are more than males in China. We cannot ascertain the reason for the gender imbalance of our sample because no official statistics have reported the gender ratio of the economically disadvantaged youths.

Last, the data was based on self-report, which may lead to the bias of conclusion. Although all questionnaires we selected have been validated in the previous studies in China, it will be helpful to integrate data from multiple informants in order to avoid the bias of self-report in future studies.

## 5. Conclusions

In conclusion, the present study examined the underlying mechanism between ostracism and depression in economically disadvantaged youths. Specifically, we can summarize that the effect of ostracism on depression is partially mediated by PsyCap in economically disadvantaged youths, and the association between ostracism and PsyCap is moderated by perceived social support among economically disadvantaged females. The study not only provides a perspective of positive psychology on revealing the pathway between ostracism and depression but also complements the social causation theory and the social selection theory. More importantly, our findings may contribute to the depression intervention targeting economically disadvantaged youths with experience of ostracism.

## Figures and Tables

**Table 1:** Mean, standard deviations, and correlations among variables.

Varibles	Mean	SD	1	2	3
1. Ostracism	28.089	6.873			
2. Psychological capital	125.031	19.663	−0.526 **		
3. Perceived support	65.735	12.384	−0.436 **	0.584 **	
4. Depression	39.232	8.128	0.623 **	−0.581 **	−0.383 **

Notes: *N* = 1207. SD, standard deviations. ** *p* < 0.001.

**Table 2:** Testing the mediation effect of psychological capital on depression.

Predictors	Model 1 (Dependent Variable: Depression)				Model 2 (Dependent Variable: Psychological Capital)				Model 3 (Dependent Variable: Depression)			
	B	SE	t	95% CI	B	SE	t	95% CI	B	SE	t	95% CI
Gender	−0.21	0.43	−0.50	[−1.04, 0.62]	−4.69	1.11	−4.22	[−6.86, −2.51]	−0.90	0.40	−2.29	[−1.68, −0.13]
Ostracism	0.74	0.03	27.51	[0.68, 0.79]	−1.53	0.07	−21.85	[−1.66, −1.39]	0.51	0.03	17.49	[0.45, 0.57]
Psychological capital									−0.15	0.01	−14.50	[−0.17, −0.13]
R^2^	0.39				0.29				0.48			
F	381.89				242.07				368.98			

Notes: B(beta), regression coefficient; SE, standard error; t, *t*-test value; CI, confidence interval; R^2^, coefficient of determination; F, analysis of Variance value.

**Table 3:** Testing the moderated mediation effect of perceived support on the association between ostracism and depression via psychological capital.

Predictors	Model 1 (Dependent Variable: Depression)				Model 2 (Dependent Variable: Psychological Capital)				Model 3 (Dependent Variable: Depression)			
	B	SE	t	95% CI	B	SE	t	95% CI	B	SE	t	95% CI
Gender	−0.12	0.42	−0.28	[−0.94, 0.70]	−5.23	0.98	−5.35	[−7.15, −3.31]	−0.90	0.40	−2.29	[−1.68, −0.13]
Ostracism	0.65	0.03	21.74	[0.59, 0.71]	−0.94	0.07	−13.47	[−1.07, −0.80]	0.51	0.03	17.49	[0.45, 0.57]
Perceived support	−0.10	0.02	−6.12	[−0.14, −0.07]	0.73	0.04	18.64	[0.65, 0.80]				
Ostracism × Perceived support	0.01	0.00	2.92	[0.001, 0.01]	−0.01	0.00	−2.95	[−0.02, −0.01]				
Psychological capital									−0.15	0.01	−14.50	[−0.17, −0.13]
R^2^	0.41				0.45				0.48			
F	206.70				243.98				368.98			

Notes: B(beta), regression coefficient; SE, standard error; t, *t*-test value; CI, confidence interval; R^2^, coefficient of determination; F, analysis of Variance value.

## Data Availability

The data presented in this study are available on request to the authors. Some variables are restricted to preserve the anonymity of study participants.

## Supplementary Materials

The following are available online at https://www.mdpi.com/article/10.3390/ijerph182111282/s1, Figure S1: Perceived social support moderates the effect of ostracism on psychological capital among female participants, Table S1: Mean, standard deviations, and correlations for all variables among male and female participants, Table S2: Testing the mediation effect of psychological capital on depression, Table S3: Testing the moderated mediation effect of perceived support on the association between ostracism and depression via psychological capital.
