# Supplementary material for: Ostracism, Psychological Capital, Perceived Social Support and Depression among Economically Disadvantaged Youths: A Moderated Mediation Model
Source: Int J Environ Res Public Health. 2021 Oct 27;18(21):11282. doi: 10.3390/ijerph182111282 (PMC8582752; doi:10.3390/ijerph182111282)
Supplement: Supplementary file 1 [file ijerph-18-11282-s001.zip › ijerph-1428360-supplementary.pdf]

Supplementary materials

Results

1 Male group

1.1 Descriptive analyses and correlation analyses

Table S1 shows means, standard deviations, and correlations for all variables among male participants. Ostracism was positively correlated with depression (r = 0.647, $p < .001$). PsyCap (r = -0.525, $p < .001$) and Perceived support (r = -0.356, $p < .001$) were negatively correlated with depression. In addition, ostracism was negatively correlated with PsyCap (r = -0.515, $p < .001$) and perceived support (r = -0.349, $p < .001$). PsyCap was positively correlated with perceived support (r = 0.584, $p < .001$).

Table S1. Mean, standard deviations, and correlations for all variables among male and female participants.

Variable	Mean		SD		1	2	3	4
	Male	Female	Male	Female				
1. Ostracism	29.003	27.786	6.758	6.888	1	-.539**	-.466**	.613**
2. Psychological capital	127.157	124.328	19.834	19.566	-.515**	1	.583**	-.608**
3. Perceived support	64.523	66.136	13.446	11.993	-.349**	.608**	1	-.391**
4. Depression	40.063	38.957	8.364	8.035	.647**	-.525**	-.356**	1

Note. The lower-left corner of the correlation matrix is correlations for all variables among male participants, and the upper right corner is those among female participants. $N = 1207$. SD, standard deviations. ** $p < 0.001$

1.2 Test of mediation

Model 4 of the SPSS macro PROCESS was utilized to test the mediating effect of PsyCap on the relationship between ostracism and depression while controlling for gender. Table S2 shows the result of the mediation analysis. Specifically, the positive predictive effect of ostracism on depression was significant (B = 0.80, t = 14.65, $p < .001$). The negative predictive effect of ostracism on PsyCap was significant (B = -1.51, t = -10.37, $p < .001$), while PsyCap had a significant negative predictive effect on depression (B = -0.11, t = -5.29, p < .001). Furthermore, the direct predictive effect of ostracism on depression was significant when the mediating variable was added (B = 0.63, t = 10.39, $p < .001$). The 95% CI of bias-corrected percentile bootstrap for the direct effect of ostracism on depression and the mediating effect of PsyCap did not include 0, which means the mediating effect was significant (Indirect effect = 0.17, SE = 0.039, 95% CI = [0.09, 0.25]). The mediating effect accounted for 20.8% of the total effect, indicating that the PsyCap played a partial mediating role in the ostracism-depression linkage.

Table S2 Testing the mediation effect of psychological capital on depression.

Predictors	Model 1 (Dependent variable: Depression)				Model 2 (Dependent variable: Psychological capital)				Model 3 (Dependent variable: Depression)			
	B	SE	*t*	95% CI	B	SE	*t*	95% CI	B	SE	*t*	95% CI

Male												
Ostracism	0.80	0.05	14.65	[0.69, 0.91]	-1.51	0.15	-10.37	[-1.80, -1.22]	0.63	0.06	10.39	[0.51, 0.75]
Psychological capital									-0.11	0.02	-5.29	[-0.15, -0.07]
R^2	0.42				0.27				0.47			
F	214.51				107.53				130.99			
Female												
Ostracism	0.72	0.03	23.34	[0.65, 0.78]	-1.53	0.08	-19.25	[-1.69, -1.37]	0.47	0.03	14.17	[0.40, 0.53]
Psychological capital									-0.16	0.01	-13.83	[-0.18, -0.14]
R^2	0.38				0.29				0.48			
F	544.59				370.41				425.22			

1.3 *Test of moderated mediation*

We hypothesized that perceived support would moderate the association between ostracism and depression. Table S3 shows the results of the moderated mediation analysis. The effect of the interaction term (ostracism × perceived support) on depression was not significant (B = -0.01, t = -0.51, p = .61).

Table S3 Testing the moderated mediation effect of perceived support on the association between ostracism and depression via psychological capital.

Predictors	Model 1 (Dependent variable: Depression)				Model 2 (Dependent variable: Psychological capital)				Model 3 (Dependent variable: Depression)			
	B	SE	*t*	95% CI	B	SE	*t*	95% CI	B	SE	*t*	95% CI
Male												
Ostracism	0.70	0.06	11.79	[0.59, 0.82]	-0.99	0.14	-7.20	[-1.26, -0.72]	0.63	0.06	10.39	[0.51, 0.75]
Perceived support	-0.11	0.03	-3.63	[-0.17, -0.05]	0.73	0.07	10.50	[0.59, 0.87]				
Ostracism × Perceived support	0.01	0.00	1.94	[-0.0001, 0.02]	0.00	0.01	-0.51	[-0.02, 0.01]				
Psychological capital									-0.11	0.02	-5.29	[-0.15, -0.07]
R^2	0.44				0.47				0.47			
F	79.06				88.88				130.99			

Female												
Ostracism	0.63	0.03	18.19	[0.56, 0.70]	-0.93	0.08	-11.46	[-1.09, -0.77]	0.47	0.03	14.17	[0.40, 0.53]
Perceived support	-0.10	0.02	-5.00	[-0.14, -0.06]	0.73	0.05	15.37	[0.63, 0.82]				
Ostracism × Perceived support	0.01	0.00	2.31	[0.0008, 0.01]	-0.02	0.01	-3.16	[-0.03, -0.01]				
Psychological capital									-0.16	0.01	-13.83	[-0.18, -0.14]
R^2	0.39				0.44				0.48			
F	195.23				88.88				425.22			

2 Female group

2.1 *Descriptive analyses and correlation analyses*

Table S1 shows means, standard deviations, and correlations for all variables among female participants. Ostracism was positively correlated with depression (r = 0.613, $p < .001$). PsyCap (r = -0.608, $p < .001$) and Perceived support (r = -0.391, $p < .001$) were negatively correlated with depression. In addition, ostracism was negatively correlated with PsyCap (r = -0.539, $p < .001$) and perceived support (r = -0.466, $p < .001$). PsyCap was positively correlated with perceived support (r = 0.583, $p < .001$).

2.2 *Test of mediation*

Model 4 of the SPSS macro PROCESS was utilized to test the mediating effect of PsyCap on the relationship between ostracism and depression. Table S4 shows the result of the mediation analysis. Specifically, the positive predictive effect of ostracism on depression was significant (B = 0.71, t = 23.34, $p < .001$). The negative predictive effect of ostracism on PsyCap was significant (B = -1.53, t = -19.25, $p < .001$), while PsyCap had a significant negative predictive effect on depression (B = -0.16, t = -13.83, $p < .001$). Furthermore, the direct predictive effect of ostracism on depression was significant when the mediating variable was added (B = 0.47, t = 14.17, $p < .001$). The 95% CI of bias-corrected percentile bootstrap for the direct effect of ostracism on depression and the mediating effect of PsyCap did not include 0, which means the mediating effect was significant (Indirect effect = 0.25, SE = 0.024, 95% CI = [0.20, 0.29]). The mediating effect accounted for 34.5% of the total effect, indicating that the PsyCap played a partial mediating role in the ostracism-depression linkage.

2.3 *Test of moderated mediation*

We hypothesized that perceived support would moderate the association between ostracism and depression. Table S3 shows the results of the moderated mediation analysis. The effect of the interaction term (ostracism × perceived support) on depression was significant (B = -0.16, t = -13.83, p = < .001). In addition, simple slope analysis showed that for youths with stronger social supports, higher ostracism was negatively associated with psychological capital (B = -1.128, t = -11.935, p = .052). However, for youths with weaker social supports, the relationship between ostracism and psychological capital was not significant (B = -0.332, t = -1.528, p = .241) (see Fig. S1).

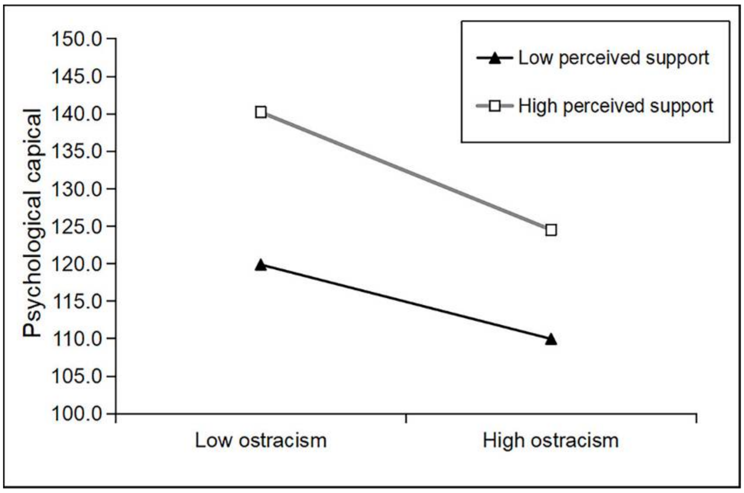

Fig. S1. Perceived social support moderates the effect of ostracism on psychological capital among female participants.
